# Draft Genome Sequences of Two Bacillus bombysepticus Strains from Drinking Water

**DOI:** 10.1128/mra.00434-23

**Published:** 2023-06-26

**Authors:** Cornelius C. Bezuidenhout, Lesego G. Molale-Tom, Rinaldo K. Kritzinger, Oluwaseyi Samuel Olanrewaju

**Affiliations:** a Unit for Environmental Sciences and Management, North-West University, Potchefstroom Campus, Potchefstroom, South Africa; DOE Joint Genome Institute

## Abstract

Human activities contribute to the contamination of drinking water sources, thereby impacting both the quality of the water and the composition of the bacterial communities present. We report the draft genome sequences of two pathogenic heterotrophic Bacillus bombysepticus strains harboring various antibiotic resistance genes; the strains were isolated from distribution water in South Africa.

## ANNOUNCEMENT

Bacillus bombysepticus is a pathogenic bacterium that infects silkworms (Bombyx mori) and causes black chest septicemia (1, 2). It is a member of the Bacillus cereus group, which includes closely related species such as Bacillus cereus, Bacillus thuringiensis, and Bacillus anthracis (2, 3). *B. bombysepticus* produces spores and parasporal crystals (1). Studies have shown that *B. bombysepticus* infection in silkworms leads to the upregulation of serine protease inhibitor genes (4). Additionally, *B. bombysepticus* has also been shown to be lethal to bees (5); however, little is known about its pathogenicity in humans.

The *B. bombysepticus* strains were isolated from a drinking water distribution source in North-West Province, South Africa, in August 2016. Strain isolation was performed on nutrient agar at 37°C for 24 h. Single colonies were picked and streaked onto nutrient agar to obtain pure isolates. The DNA of each pure isolate was extracted using the chemagic DNA bacteria kit (PerkinElmer, Germany), following the manufacturer’s protocol.

DNA libraries were prepared using the Nextera XT kit (Illumina Inc., San Diego, CA) according to the instructions provided by the manufacturer and sequenced using the Illumina MiSeq 300 platform with paired-end reads at the North-West University sequencing facility.

The generated raw paired-end fastq reads (2 × 300 bp) were quality checked using FastQC v.0.11.7 (6); low-quality bases were trimmed using Trimmomatic v.0.39 (7), and the data quality was rechecked using FastQC v.0.11.7 (6). The cleaned reads were assembled using SPAdes v.3.15.5 (8). QUAST v.5.0.2 (9) was used to evaluate the quality of the genome assembly. The completeness and contamination were assessed using CheckM v.1.1.6 (10). The assembled draft genomes were annotated using the NCBI Prokaryotic Genome Annotation Pipeline (PGAP) v.6.5 (11) and the Rapid Annotations using Subsystems Technology (RAST) pipeline (12), and a summary report is presented in Table 1. The annotated genomes were assessed against the Genome Taxonomy Database (GTDB) using GTDB-Tk software v.1.7.0 (13). Default parameters were used for all software except where otherwise noted.

**TABLE 1:** Annotation and genome assembly statistics of the reported strains

Parameter	Data for strain:	
	2D2-5	2D3-3
No. of raw reads	3,026,970	2,715,060
No. of filtered reads	2,675,072	2,425,846
Total size of raw reads (bp)	767,871,918	701,880,979
Total size of filtered reads (bp)	606,551,364	555,899,372
Coverage (×)	130.7	94.6
Completeness (%)	99.43	99.43
Contamination (%)	0.7	1.53
GenBank accession no. for FastANI reference sequence	GCF_006384875.1	GCF_006384875.1
Closest FastANI match (ANI [%])	*Bacillus bombysepticus* (97.75)	*Bacillus bombysepticus* (97.78)
Annotation data (RAST/PGAP)		
Assembly size (bp)	5,969,053/5,969,053	5,968,101/5,968,101
GC content (%)	34.8/34.8	34.8/34.8
*N*_50_ (bp)	335,855/335,855	333,245/333,245
*L*_50_	5/5	6/6
No. of protein-encoding genes	NA^*a*^/5,834	NA/5,816
No. of contigs	71/71	71/71
No. of subsystems	339/NA	340/NA
No. of coding sequences	6,193/6,012	6,198/5,995
No. of RNAs	107/119	107/118
No. of rRNAs	NA/42	NA/40
No. of tRNAs	NA/93	NA/93
No. of ncRNAs^*b*^	NA/5	NA/5

a NA, not available.

b ncRNAs, noncoding RNAs.

Genome assembly statistics and annotation features are presented in Table 1. In conclusion, these new genomes will contribute further insight into the biology and diversity of this bacterial species.

### Data availability.

The whole-genome shotgun project for strains 2D2-5 and 2D3-3 has been deposited at DDBJ/ENA/GenBank under accession numbers JASDAQ000000000 and JASDAR000000000; the versions described in this paper are versions JASDAQ010000000 and JASDAR010000000, respectively. The raw reads for 2D2-5 and 2D3-3 are available under BioProject accession numbers PRJNA968030 and PRJNA968031, respectively, and the BioSample accession numbers are SAMN34894822 and SAMN35004863, respectively. The sequence data obtained in this work have been deposited in the NCBI Sequence Read Archive under accession numbers SRR24490058 and SRR24491038 (2D2-5 and 2D3-3, respectively).
